# Validity and reliability of the Japanese versions of cognitive and behavioral scales for irritable bowel syndrome

**DOI:** 10.1186/s13030-022-00244-3

**Published:** 2022-07-23

**Authors:** Nagisa Sugaya, Yoshitoshi Tomita, Misako Funaba, Hiroshi Iida, Kentaro Shirotsuki, Fumiyuki Chin Gardner, Toshinari Odawara, Tetsuya Ando, Masahiko Inamori

**Affiliations:** 1 Unit of Public Health and Preventive Medicine, School of Medicine, Yokohama City University, 3-9 Fukuura, Kanazawa-ku, Yokohama, 236-0004 Japan; 2 Department of Psychosomatic Medicine, National Center Hospital, National Center of Neurology and Psychiatry, 4-1-1 Ogawahigashi, Kodaira, Tokyo, 187-8553 Japan; 3 Department of Behavioral Medicine, National Institute of Mental Health, National Center of Neurology and Psychiatry, 4-1-1 Ogawahigashi, Kodaira, Tokyo, 187-8553 Japan; 4 Department of Medical Education, School of Medicine, Yokohama City University, 3-9 Fukuura, Kanazawa-ku, Yokohama, 236-0004 Japan; 5 Faculty of Human Sciences, Musashino University, 3-3-3 Ariake, Koto-ku, Tokyo, 135-8181 Japan; 6 Department of Pediatrics, The Pennsylvania State University, 500 University Drive, Hershey, PA 17033 USA; 7 Health Management Center, Yokohama City University, 22-2 Seto, Kanazawa-ku, Yokohama, 236-0027 Japan; 8 Department of Psychosomatic Medicine, Narita Hospital, International University of Health and Welfare, 852 Hatakeda, Narita, Chiba 286-8686 Japan

**Keywords:** Irritable bowel syndrome, Cognitive scale for functional bowel disorders, Irritable bowel syndrome-behavioral responses questionnaire, Japanese version

## Abstract

**Background:**

The Cognitive Scale for Functional Bowel Disorders (CS-FBD) and Irritable Bowel Syndrome-Behavioral Responses Questionnaire (IBS-BRQ) are a useful measures to assess cognitive-behavioral aspects in individuals with IBS. This study aimed to confirm the reliability and validity of the Japanese versions of the CS-FBD (CS-FBD-J) and IBS-BRQ (IBS-BRQ-J).

**Methods:**

Participants comprised 192 students and 22 outpatients diagnosed with irritable bowel syndrome (IBS). There were 76 students who met the diagnostic criteria for IBS and two students who received treatment for IBS. Participants completed questionnaires containing the CS-FBD-J, IBS Severity Index (IBS-SI), Visceral Sensitivity Index (VSI), 24-item Dysfunctional Attitudes Scale (DAS-24), Hospital Anxiety and Depression Scale (HADS), and Social Adaptation Self-evaluation Scale (SASS).

**Results:**

Our exploratory factor analysis revealed that the CS-FBD-J had a unidimensional factor structure and that the factor loadings for two of the 25 items were less than 0.4. The IBS-BRQ-J had a two-factor structure, and the factor loadings for eight of the 26 items were less than 0.4. The confirmatory factor analysis for the 18-item version of IBS-BRQ-J showed that the model fit indices were not sufficient. The CS-FBD-J and IBS-BRQ-J had significant, moderate correlations with the IBS-SI and VSI in the IBS and control groups. Correlation between the DAS-24 and the CS-FBD-J was not significant. The CS-FBD-J and IBS-BRQ-J were significantly correlated to the HADS and SASS (IBS-BRQ-J) only in the IBS group. The scores of CS-FBD-J and IBS-BRQ-J showed significant group differences between the IBS patient group, non-patient IBS group, and control group. The internal consistencies of the CS-FBD-J and IBS-BRQ-J were high. The item-total correlation analysis for the CS-FBD-J and IBS-BRQ-J showed that the correlations between each item and the total score were significant.

**Conclusion:**

This study confirmed the reliability and validity of the 23-item version of the CS-FBS-J and the 18-item version of the IBS-BRQ-J with the deletion of items with low factor loadings. Regarding the IBS-BRQ-J, two factor structures were confirmed (factor 1: behavior obsessed with abdominal symptoms, factor 2: avoidance of abdominal symptoms and associated difficulties) although the model fit of the structure needs further study.

**Supplementary Information:**

The online version contains supplementary material available at 10.1186/s13030-022-00244-3.

## Background

Irritable bowel syndrome (IBS) is a functional gastrointestinal disorder characterized by persistent chronic abdominal pain and disturbance of bowel movements [1]. It is a typical psychosomatic disorder of the digestive system that is exacerbated by psychological stress. Our previous study [2] reported that the cognitive-emotion process is related to the aggravation of anxiety in individuals with IBS; severe anxiety sensitivity in individuals with IBS related to their symptom-related cognition and the altered cognition associated with increased anxiety can lead to the development of a disabling condition, including avoidant behavior. Behavioral problems also related to gastrointestinal symptoms play an important role in the pathophysiology of IBS. The majority of participants with IBS are reported to have a high level of impairment or avoidance of daily activities [3].

Based on the above characteristics of individuals with IBS, many previous studies have reported the effect of cognitive-behavioral (CBT) or cognitive therapy on the abdominal symptoms and emotional distress of patients with IBS [4, 5]. Furthermore, various measures explicitly developed for IBS, such as the Visceral Sensitivity Index (VSI [6]) and the IBS-QOL [7], have been used as outcomes of such IBS intervention studies, including gastrointestinal symptoms and general anxiety and depression. Additionally, it is essential in CBT to assess the cognitive-behavioral aspects of IBS appropriately because these are variables directly targeted for such intervention.

The Cognitive Scale for Functional Bowel Disorders (CS-FBD [8]) is a useful measure to assess maladaptive cognitions, focusing on functional bowel disorders related to negative thoughts, perfectionism, and social desirability. The CS-FBD is a valid and reliable scale that can be used as an outcome measure in evaluating the efficacy of different forms of psychotherapeutic intervention for functional bowel disorders (e.g., IBS, functional diarrhea/constipation), and can also serve as a helpful assessment tool for health professionals working with patients diagnosed with IBS [8]. CS-FBD has been used in many previous studies, including clinical trials of CBT [9–14]. The VSI [6] has been developed to assess gastrointestinal-specific anxiety, cognitive, affective, and behavioral responses to fear of GI sensations, symptoms, and the context in which they occur, and it has been translated into Japanese [15]. The CS-FBD focuses on maladaptive cognition that is not specific to anxiety, and its content is differentiated from the VSI. CS-FBD items were generated from the diaries of automatic thought records in patients with functional gastrointestinal disorders, and included a variety of themes: bowel performance anxiety, out of control, perfectionism, anger/frustration, self-efficacy, social approval, embarrassment/shame, heightened sensitivity to social rules and norms, and self-nurturance. Unlike the VSI, the CS-FBD is a "cognition"-specific scale. It has the advantage of precisely assessing the role of cognition in the cognitive-behavioral model of IBS and the degree of cognitive transformation achieved in CBT. The CS-FBD was utilized along with the VSI in previous studies (e.g., [13]).

Additionally, the irritable bowel syndrome-behavioral responses questionnaire (IBS-BRQ) [16] was developed to assess the IBS-specific pattern of avoidance and control behavior, and has been validated for individuals with IBS and has convergent validity and good test-retest reliability. IBS-BRQ has been used in many previous studies [14, 17–22]. Bonnert et al. [17] reported that changes in IBS-related unhelpful behavior assessed by the IBS-BRQ [16] mediated the effect of exposure-based Internet-CBT on gastro-intestinal (GI) symptoms. The decrease in avoidance behavior explained a large portion (67%) of the total treatment effect. They also observed a unidirectional relation over time between avoidance behavior and GI symptoms, corroborating a causal effect. Additionally, Windgassen et al. [22] found that an increased avoidance behavior score, one of the subscales of the IBS-BRQ, was associated with an increased frequency of diarrhea symptoms, and that a higher control behavior score, another subscale of the IBS-BRQ, was associated with a higher frequency of hard/lumpy stools.

Hence, the CS-FBD and IBS-BRQ may contribute to develop cognitive-behavioral model of IBS and to refining the assessment of effect of psychological treatment for IBS. However, the Japanese versions of CS-FBD and IBS-BRQ have not been fully assessed.

The purpose of this study is to confirm the reliability and validity of the Japanese version of the CS-FBD (CS-FBD-J) [Study 1] and IBS-BRQ (IBS-BRQ-J) [Study 2].

## Methods

### Participants

Participants were recruited between September 2013 and March 2021. They were outpatients who were diagnosed with IBS in the gastroenterology and psychosomatic medicine departments of hospitals in Tokyo and Kanagawa and college students, including patients with IBS who met the diagnostic criteria (Rome III criteria) and others who did not meet the diagnostic criteria. The present study was approved by the ethics committee of Yokohama City University (B130905050) and the National Center of Neurology and Psychiatry (A2018-046). Written informed consent was obtained from all participants.

### Measures

#### Cognitive Scale for Functional Bowel Disorders translated into Japanese

The Cognitive Scale for Functional Bowel Disorders (CS-FBD) [8] includes 25 items that measure maladaptive cognition related to abdominal symptoms, with responses ranging from 1 (*strongly disagree*) to 7 (*strongly agree*). The total score for this scale ranges from 25 to 175. One of the authors (N.S.), who is a psychologist knowledgeable about functional gastrointestinal disorders and psychosomatic medicine, translated the original version into Japanese. Another author (F.G.) who is bilingual in English and Japanese, back-translated the Japanese items into English. We compared the results of the back translation to the original version and confirmed agreement among the researchers about the nuances.

#### Irritable bowel syndrome-behavioral responses questionnaire translated into Japanese

The IBS-BRQ [16] includes 26 items with responses ranging from 1 (never) to 7 (always). It has two factors: avoidance behavior scale and control behavior scale. The IBS-BRQ was translated into Japanese using a standard method. One of the authors (N.S.), who is a psychologist well versed in functional gastrointestinal disorders and psychosomatic medicine, translated the original version into Japanese. Another author (F.G.) who is bilingual in English and Japanese, back-translated the Japanese items into English. We compared the results of the back translation to the original version and confirmed the agreement among the researchers about the nuances.

#### Japanese version of the Rome III modular questionnaire (only items related to IBS)

The Rome III Diagnostic Criteria for Functional Gastrointestinal Disorders [23] have been widely used to define IBS. Rome IV had not been published at the start of this investigation. Using the Rome III modular questionnaire [24, 25], the presence of IBS was determined if the participants had abdominal pain or discomfort for at least three weeks (at least once per week) in the previous three months and two of the three following symptoms: (1) pain or discomfort that improves or stops after a bowel movement, (2) a change in the number of bowel movements when the pain or discomfort starts, and (3) either softer or harder stools than usual when the pain or discomfort starts. There are four defined subtypes of IBS. The diarrhea-predominant IBS (IBS-D) group includes individuals with loose, mushy, or watery stools more than 25% of the time and no or rare hard or lumpy stools. In constipation-predominant IBS (IBS-C), individuals pass hard or lumpy stools more than about 25% of the time and no or rare loose, mushy, or watery stools. The mixed type IBS (IBS-M) group includes individuals with loose, mushy or watery stools and hard or lumpy stools more than 25% of the time. Finally, individuals with unclassifiable IBS (IBS-U) pass rare loose, mushy or watery stools or rare hard or lumpy stools or none at all.

#### IBS severity index

The IBS Severity Index (IBS-SI) [26, 27] was used to assess the severity of gastrointestinal symptoms. This scale consists of 5 items: abdominal pain (2 items), abdominal distension, bowel movements, and quality of life, and the total score ranges from 0 to 500. The IBS-SI classifies the severity of IBS as mild (75–174), moderate (175–299), or severe (300–500) based on clinical observations of patients with IBS.

#### Japanese version of the Visceral Sensitivity Index

The Visceral Sensitivity Index (VSI) [6, 15] is a self-reported measure of gastrointestinal symptom-specific anxiety in patients with IBS. The VSI includes 15 items with responses ranging from 1 (*strongly agree*) to 6 (*strongly disagree*) and scores ranging from 0 to 5.

#### Japanese version of the 24-item Dysfunctional Attitudes Scale

The 24-item Dysfunctional Attitudes Scale (DAS-24) [28, 29] is a self-report scale for depression schemata with responses ranging from 1 (*strongly disagree*) to 7 (*strongly agree*). The DAS-24 contains three factors: “achievement,” “self-control,” and “dependency.” The achievement subscale consists of eight items, for example, “If I fail partly, it is as bad as being a complete failure.” The dependency subscale consists of eight items, for example, “I am nothing if a person I love doesn’t love me.” The self-control subscale consists of eight items, for example, “A person should do well at everything he undertakes.”

#### Hospital Anxiety and Depression Scale

The Hospital Anxiety and Depression Scale (HADS) [30, 31] is a self-report questionnaire for anxiety and depression. The HADS includes 14 questions on a 4-point scale, consisting of an anxiety subscale with seven items and a depression subscale with seven items. This psychometric instrument was chosen because all its items refer solely to an emotional state and do not consider somatic symptoms.

#### Japanese version of the Social Adaptation Self-evaluation Scale

The Social Adaptation Self-Evaluation Scale (SASS) [32, 33] was developed to assess social impairment caused by depression. This scale has 21 items, with scores ranging from 0 to 3. Lower SASS score indicates impaired social adaptation.

### Procedure

#### Patients

Patients with IBS were given written and oral explanations about the purpose of this study, the protection of their personal information, and that participation was voluntary. Patients with IBS who agreed to participate provided informed consent. Participants then completed questionnaires containing the physical and psychological scales mentioned above.

#### College students

The researchers recruited college students after class hours or via the university’s online bulletin board. Participants recruited by the latter method were limited to those who met the diagnostic criteria for IBS. Informed consent was obtained using the same procedures as for patients.

### Statistical analysis

The factor structure of the CS-FBD-J and the IBS-BRQ-J was confirmed by exploratory factor analysis. For the IBS-BRQ-J, confirmatory factor analysis using the Structure Equation Model (SEM) was conducted. In the SEM, assessment of model fit was based on multiple indicators; Goodness-of-Fit Index (GFI), Adjusted goodness-of-Fit Index (AGFI), Comparative fit index (CFI), and Root-mean-square error of approximation (RMSEA). Construct validity was confirmed using Pearson’s correlation coefficient analysis between the CS-FBD-J and scores on the IBS-SI, VSI, DAS, and HADS and between the IBS-BRQ-J and scores on the IBS-SI, the item of disruption to daily life (IBS-SI), VSI, HADS, and SASS in the IBS and control groups. Criterion-related validity was confirmed using analysis of covariance to compare the CS-FBD-J score and the IBS-BRQ-J between the IBS patient group, the non-patient IBS group, and the control group after adjusting age, and a post-hoc test with Bonferroni’s method was employed to test the differences between groups. We compared these three groups to investigate criterion-related validity more precisely because the IBS patient and non-patient IBS groups were expected to have different severity of psychosomatic symptoms. The internal consistency reliability of the CS-FBD-J and the IBS-BRQ-J was determined using Cronbach’s α. The correlations between each item and the total score of the CS-FBD-J were used to conduct item-total correlation analysis. The comparisons of sex ratio between the IBS patient, non-patient IBS, and control groups were conducted using the *χ*^2^ test. One-way analysis of variance was applied to compare age between these groups. Analysis of covariance was used to compare IBS-SI, the item of disruption to daily life (IBS-SI), VSI, HADS, and SASS between these groups after adjusting age. The significance level was set at less than 5%. Data analyses were performed using SPSS software (version 25.0; IBM Corp., NY, USA).

## Results

### Descriptive results

**Fig. 1:**
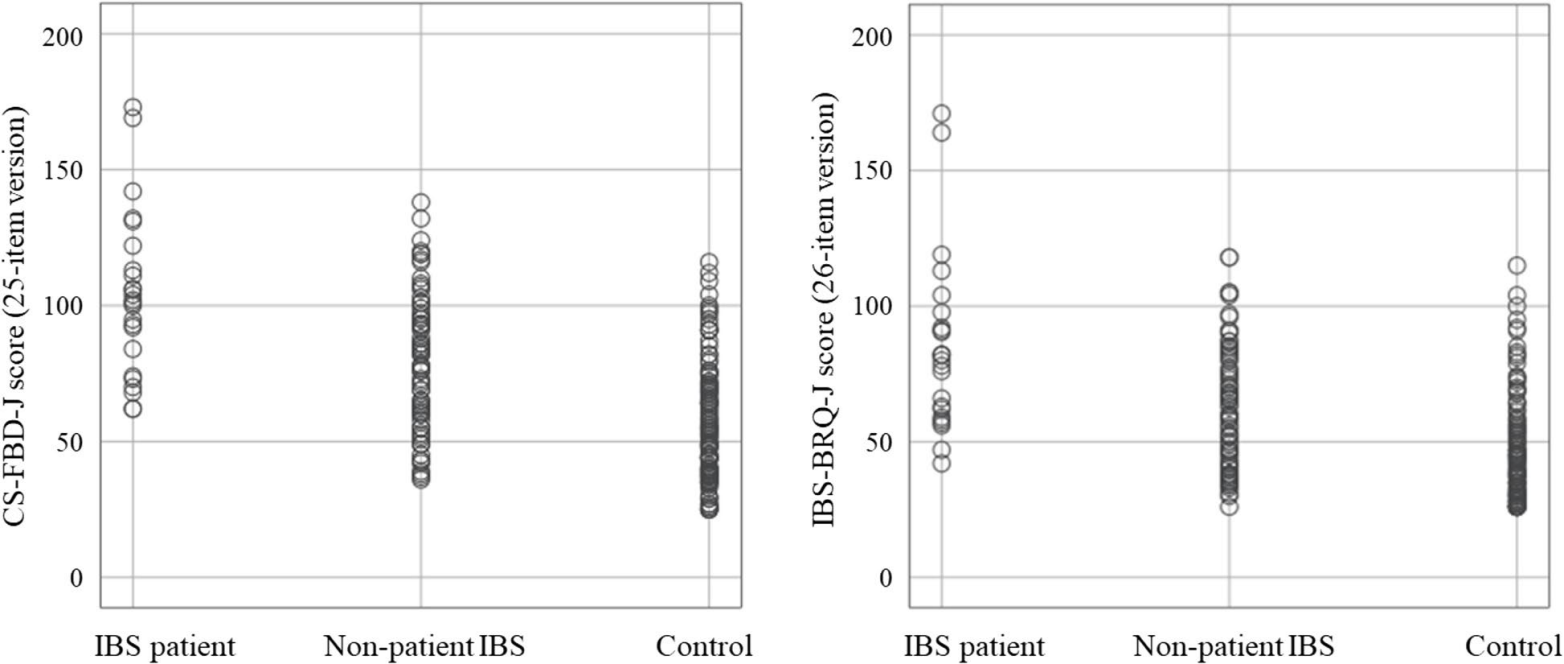
Plots of the CS-FBD-J and IBS-BRQ-J scores of IBS patient, non-patient IBS, and control groups. IBS: irritable bowel syndrome. CS-FBD-J: Japanese version of Cognitive Scale for Functional Bowel Disorders. IBS-BRQ: Irritable Bowel Syndrome-Behavioral Responses Questionnaire

Table 1 presents the descriptive results for the participants. In our study, 203 college students were enrolled, and 192 students (53 male and 139 female, 20.2±3.0 yrs.) provided valid data. There were 76 students who met the diagnostic criteria for IBS, and two students received treatment for IBS. Twenty-two outpatients who were diagnosed with IBS in the gastroenterology or psychosomatic medicine departments of hospitals in Tokyo and Kanagawa (17 male and 5 female, 38.0±13.0 yrs.) provided valid data. Eleven participants who received treatment for IBS received psychiatric medication. Two IBS patients did not meet the presence of abdominal discomfort or pain for at least six months criterion of IBS. This may have been due to a period of symptom resolution with treatment. However, because they met all other criteria during the survey period, they were included in the IBS patient group. The IBS group (IBS patients and non-IBS patients) included 28 participants with diarrhea-predominant IBS (IBS-D), 9 with constipation-predominant IBS (IBS-C), 55 with mixed-type IBS (IBS-M), and 6 with unclassifiable IBS (IBS-U). There was a significant difference of sex ratio between the IBS patient, non-patient IBS, and control groups (*χ*^2^ = 17.86, *p* < 0.001, Cramer’s *V* = 0.289). The prevalence of male sex was higher among IBS patients than in the other groups. Table 2 shows the differences in each variable between the IBS patient (*n* =24), non-patient IBS (*n* =74), and control groups (*n* =116) after adjusting for age. Figure 1 shows the plot of the CS-FBD-J score and the IBS-BRQ-J score in each group. There was a significant difference of age between groups (*F* [2, 211] = 102.88, *p* < 0.001). The IBS patient group had a higher mean age than other groups. After adjusting for age, there were significant group effects in the IBS-SI, disruption to daily life, VSI, and SASS. Multiple comparison analysis showed higher scores of the IBS-SI and disruption to daily life in the IBS patient and non-patient groups than in the control group. There was a significant difference in the VSI between all groups. The SASS score of the IBS patients was lower than that of the non-patient IBS and control groups. Although there was a significant group effect of the DAS, multiple comparison analysis did not show any significant differences among the groups.

**Table 1:** Descriptive results of the study participants

		*N*(%)			
	Total	Male		Female	
IBS patient	24	17	(70.8)	7	(29.2)
Non-patient IBS	74	21	(28.4)	53	(71.6)
Control	116	32	(27.6)	84	(72.4)
IBS-D	28	13	(46.4)	15	(53.6)
IBS-C	9	1	(11.1)	8	(88.9)
IBS-M	55	22	(40.0)	33	(60.0)
IBS-U	6	2	(33.3)	4	(66.7)

*IBS* Irritable bowel syndrome

*IBS-D* Diarrhea-predominant IBS

*IBS-C* Constipation-predominant IBS

*IBS-M* Mixed-type IBS

*IBS-U* Unclassifiable IBS

**Table 2:** Comparison of each variable between IBS patient, non-patient IBS, and control groups after adjusting for age

	Mean (*SD*)								
	IBS patient (*n* = 24)		Non-patient IBS (*n* = 74)		Control (*n* = 116)		*F*	*p*	*η*_*p*_^2^
CS-FBD (25 items)	103.5	(30.2)	78.6	(25.1)	57.1	(21.9)	29.01	< 0.001	0.216
CS-FBD (23 items)	93.7	(29.2)	69.8	(24.6)	49.4	(20.7)	28.40	< 0.001	0.213
IBS-BRQ (26 items)	84.7	(32.4)	62.4	(22.8)	46.7	(19.8)	21.91	<0.001	0.173
IBS-BRQ (18 items)	60.2	(24.0)	42.2	(17.0)	30.0	(14.2)	24.50	<0.001	0.189
IBS-SI	193.3	(109.7)	193.9	(80.7)	116.1	(86.6)	18.96	< 0.001	0.153
Disruption to daily life (IBS-SI)	56.9	(30.4)	43.4	(25.3)	25.4	(27.0)	11.58	< 0.001	0.101
VSI	52.4	(14.7)	23.2	(19.8)	13.6	(17.0)	22.69	< 0.001	0.197
DAS	92.1	(24.4)	93.1	(24.5)	87.3	(20.7)	3.50	0.032	0.033
HADS (Anxiety)	7.4	(4.8)	7.3	(3.6)	6.4	(3.8)	2.73	0.067	0.026
HADS (Depression)	5.3	(4.8)	4.8	(3.0)	4.9	(3.8)	0.21	0.811	0.002
HADS (Total)	12.7	(9.1)	12.1	(6.0)	11.3	(7.0)	1.08	0.342	0.010
SASS	29.9	(7.8)	35.9	(6.4)	37.4	(6.5)	6.24	0.002	0.058

*IBS* irritable bowel syndrome

*CS-FBD-J* Japanese version of Cognitive Scale for Functional Bowel Disorders

*IBS-BRQ* Irritable Bowel Syndrome-Behavioral Responses Questionnaire

*IBS-SI* IBS Severity Index

*VSI* Visceral Sensitivity Index

*DAS-24* 24-item Dysfunctional Attitudes Scale

*HADS* Hospital Anxiety and Depression Scale

*SASS* Social Adaptation Self-evaluation Scale

### Study 1 (validity and reliability of the CS-FBD-J)

#### Factor analysis

Exploratory factor analysis (using maximum likelihood estimation and promax rotation) without assigning the number of factors revealed the presence of five components with eigenvalues exceeding 1. However, inspection of the scree plot revealed a break after the first component. Therefore, we decided to retain a unidimensional factor structure for further investigation (Table 3). Factor analysis showed that the factor loadings for two items (items 6 and 18) were less than 0.4. After excluding these items from the analysis, the factor loadings for all items were above 0.4, and a single factor of the 23-item version accounted for 45.85% of the total variance.

**Table 3:** Results of the item-total analysis and factor analysis

	25-item version		23-item version	
Items	Item-total correlation	Factor loadings	Item-total correlation	Factor loadings
19. Not take advantage of opportunities due to bowel problems	0.720	0.785	0.727	0.786
16. Concern about lasting through events	0.802	0.781	0.817	0.782
23. Worry about losing control of bowels in public	0.810	0.770	0.812	0.768
12. Bowel problems interfere with feeling good about myself	0.761	0.769	0.763	0.769
14. Can’t concentrate due to pain	0.767	0.766	0.780	0.767
13. Worry about my bowel symptoms on a trip	0.789	0.763	0.800	0.763
9. I feel very down about my bowel symptoms	0.712	0.756	0.730	0.759
7. Frustrated by bowel symptoms	0.722	0.754	0.734	0.755
5. Bowel symptoms are agony	0.763	0.733	0.763	0.732
8. Pain will never go away	0.700	0.724	0.717	0.727
20. Symptoms make me feel out of control	0.686	0.724	0.693	0.726
11. Worry about not finding a bathroom when I need one	0.774	0.714	0.776	0.712
2. I’m always sick with bowel problems	0.686	0.705	0.688	0.705
4. Can’t function normally when sick with bowel problems	0.731	0.696	0.735	0.695
3. Symptoms are too much to handle	0.692	0.693	0.698	0.696
21. Bowel symptoms in restaurant	0.639	0.635	0.653	0.636
22. With frequent bathroom visits, others think something is wrong	0.668	0.627	0.663	0.622
15. Embarrassing to keep going to bathroom	0.611	0.581	0.607	0.576
25. Must get home when 1 have symptoms	0.553	0.541	0.563	0.542
1. Not getting to bathroom in time	0.483	0.490	0.489	0.491
17. Being late upsets me	0.567	0.480	0.541	0.472
24. Feeling guilt if I nurture myself	0.431	0.452	0.433	0.450
10. Passing gas in public	0.471	0.416	0.473	0.414
18. Hate making a fool of myself	0.389	0.313	-	-
6. Do my absolute best at everything	0.323	0.262	-	-
	Eigenvalue	10.72	Eigenvalue	10.54
	Variance (%)	42.87%	Variance (%)	45.85%

#### Construct validity

The results of the correlation analysis between the CS-FBD-J and other variables in the IBS and control groups confirmed the construct validity (Table 4). The scores of the 25-item and 23-item versions of CS-FBD-J had significant moderate correlations with the IBS-SI and VSI scores in all participants, the IBS group, and the control group. The DAS-24 score was significantly correlated with the 25-item version of CS-FBD-J in both the IBS and control groups, but the correlations were low. Moreover, correlations between the 23-item version of CS-FBD-J and DAS-24 did not reach a significant level. Regarding the HADS, although the 25-item and 23-item versions of the CS-FBD-J in the IBS group were significantly correlated with the HADS-anxiety and depression scores, their intercorrelations in the control group were not significant.

**Table 4:** Correlations between the CS-FBD-J (25-item and 23-item version) and other variables

25-item version		IBS-SI	VSI	DAS-24	HADS (Anxiety)	HADS (Depression)	HADS (Total)
Total	*r*	0.617	0.625	0.249	0.335	0.219	0.305
	*p*	< 0.001	< 0.001	< 0.001	< 0.001	0.001	< 0.001
IBS group	*r*	0.569	0.553	0.213	0.455	0.361	0.444
	*p*	< 0.001	< 0.001	0.035	< 0.001	< 0.001	< 0.001
Control group	*r*	0.488	0.515	0.224	0.162	0.144	0.169
	*p*	< 0.001	< 0.001	0.017	0.084	0.127	0.073
23-item version		IBS-SI	VSI	DAS-24	HADS (Anxiety)	HADS (Depression)	HADS (Total)
Total	*r*	0.620	0.632	0.221	0.336	0.222	0.307
	*p*	< 0.001	< 0.001	0.001	< 0.001	0.001	< 0.001
IBS group	*r*	0.574	0.557	0.191	0.450	0.357	0.440
	*p*	< 0.001	< 0.001	0.059	< 0.001	< 0.001	< 0.001
Control group	*r*	0.492	0.531	0.179	0.167	0.150	0.175
	*p*	< 0.001	< 0.001	0.058	0.075	0.111	0.062

*IBS* irritable bowel syndrome

*CS-FBD-J* Japanese version of Cognitive Scale for Functional Bowel Disorders

*IBS-SI* IBS Severity Index

*VSI* Visceral Sensitivity Index

*DAS-24* 24-item Dysfunctional Attitudes Scale

*HADS* Hospital Anxiety and Depression Scale

#### Criterion-related validity

We compared the scores of the 25-item and 23-item versions of the CS-FBD-J between the IBS patient, non-patient IBS, and control groups after adjusting for age to confirm the criterion-related validity (Table 2). There were significant group effects in both versions of the CS-FBD-J, and multiple comparison analysis showed significant differences between all groups (i.e., IBS patient > non-patient IBS > control).

#### Reliability

Cronbach’s α was calculated to estimate the reliability of the CS-FBD-J. The internal consistencies of the 25-item and 23-item versions (excluding items 6 and 18) in all participants, the IBS group, and the control group were high (25-item version: all participants: *α* = 0.94, IBS group: *α* = 0.94, control group: *α* = 0.92; 23-item version: all participants: *α* = 0.95, IBS group: *α* = 0.94, control group: *α* = 0.92).

The item-total correlation analysis for the 25-item and 23-item versions of the CS-FBD-J (Table 3) showed that the correlations between each item and the total score were significant (25-item version: *p*s < 0.001, *r*s = 0.323–0.810; 23-item version: *p*s < 0.001, *r*s = 0.433–0.812).

### Study 2 (validity and reliability of the IBS-BRQ-J)

#### Factor analysis

Exploratory factor analysis (using maximum likelihood estimation and promax rotation) without assigning the number of factors revealed the presence of six components with eigenvalues exceeding 1. However, inspection of the scree plot revealed a break after the second component. Therefore, we decided to retain a two-factor structure for further investigation (Table 5). Factor analysis showed that the factor loadings for eight items (items 1, 2, 8, 9, 10, 11, 23, and 24) were less than 0.4. After excluding these items from the analysis, the factor loadings for all items were above 0.4, and two factors of the 18-item version accounted for 43.84% and 51.51 % of the total variance, respectively.

**Table 5:** Results of the item-total analysis and factor analysis

	26-item version			18-item version		
		Factor loadings			Factor loadings	
Items	Item-total correlation	Factor 1	Factor 2	Item-total correlation	Factor 1	Factor 2
13. I avoid making plans in case I have problems with my IBS	0.706	**0.936**	-0.075	0.699	**0.950**	-0.083
12. I avoid going out in case I have problems with my IBS	0.726	**0.894**	-0.020	0.721	**0.909**	-0.033
26. I avoid staying away from home overnight in case my IBS flares up	0.579	**0.859**	-0.159	0.595	**0.847**	-0.130
21. I avoid certain social situations (e.g., restaurants) because of my IBS	0.655	**0.794**	-0.016	0.668	**0.758**	0.035
20. I avoid certain work situations (e.g., meetings) because of my IBS	0.736	**0.794**	0.086	0.752	**0.758**	0.146
14. I carry other items (e.g., wet wipes, sanitary towels, spare underwear) in case my IBS flares up	0.617	**0.683**	-0.110	0.608	**0.682**	-0.121
17. I avoid sex in case my IBS flares up (and causes embarrassment)	0.559	**0.682**	-0.161	0.551	**0.716**	-0.207
15. I take medication (e.g., before going out) just in case my IBS flares up	0.683	**0.665**	0.017	0.704	**0.599**	0.100
22. I avoid certain foods (e.g., dairy products, spicy foods) because of my IBS	0.665	**0.626**	0.114	0.667	**0.586**	0.155
19. I ask for reassurance about my IBS	0.690	**0.610**	0.267	0.706	**0.559**	0.341
16. I carry medication with me in case my IBS flares up	0.693	**0.516**	0.125	0.725	**0.439**	0.221
24. When I have diarrhea I do things to ease it (e.g. take prescribed medication, take alternative medication)	0.623	**0.436**	0.122		-	-
10. I avoid certain foods when I have bowel problems	0.600	**0.301**	0.211		-	-
9. I avoid exercise when I have stomach pains	0.511	**0.290**	0.151		-	-
11. I wear baggy clothing when my stomach feels bloated or distended	0.463	**0.252**	0.112		-	-
6. I spend more time on the toilet than I ideally would like	0.632	-0.087	**0.733**	0.655	-0.068	**0.694**
4. After opening my bowels I check for blood	0.453	-0.170	**0.703**	0.487	-0.167	**0.686**
5. After opening my bowels I check my stool for abnormalities	0.526	-0.170	**0.701**	0.574	-0.188	**0.712**
3. I strain when opening my bowels	0.635	0.107	**0.650**	0.640	0.127	**0.603**
25. I am constantly aware of my stomach	0.752	0.117	**0.639**	0.759	0.032	**0.733**
7. I often go to the toilet to open my bowels and then do not pass anything	0.564	0.019	**0.545**	0.581	0.053	**0.482**
18. When I go out, I make sure I know where the nearest toilet is	0.741	0.366	**0.441**	0.746	0.295	**0.525**
8. I often go to the toilet to pass water and find I open my bowels	0.476	0.125	**0.426**		-	-
1. I eat specific foods to help me open my bowels more	0.481	0.022	**0.399**		-	-
2. I eat specific foods to help me open my bowels less	0.491	0.240	**0.393**		-	-
23. After I open my bowels, I wipe more than I would like	0.609	0.264	**0.390**		-	-
	Eigenvalue	9.63	1.57	Eigenvalue	7.89	1.38
	Variance (%)	37.03%	43.06%	Variance (%)	43.84%	51.51%
	Cronbach’s α =0.93			Cronbach’s α = 0.92		

**Fig. 2:**
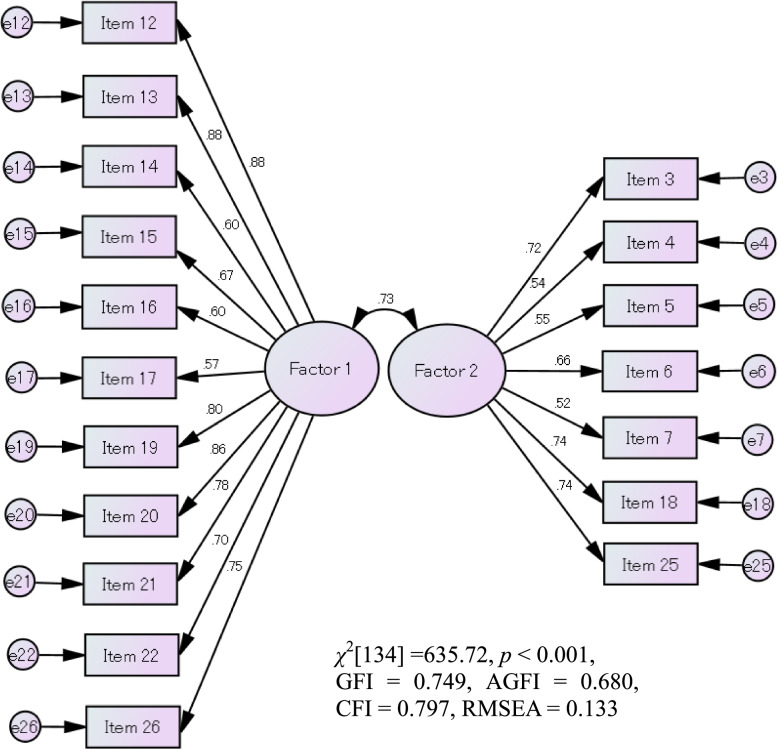
The results of the SEM. GFI: Goodness-of-Fit Index, AGFI: Adjusted goodness-of-Fit Index, CFI: Comparative fit index, RMSEA: Root-mean-square error of approximation

In the results of the SEM for the 18-tem version (Fig. 2), the model fit indices were not sufficient (GFI = 0.749, AGFI = 0.680, CFI = 0.797, RMSEA = 0.133).

#### Construct validity

The results of the correlation analysis between IBS-BRQ-J and other variables in the IBS and control groups were examined for construct validity. Regarding both the 26-item and 18-item versions of IBS-BRQ-J, the scores of IBS-SI, disruption to daily life, and VSI showed significant, positive correlations with the IBS-BRQ-J scores in both the IBS and control groups, while the scores of anxiety (HADS), depression (HADS), and SASS were significantly correlated to the IBS-BRQ-J score only in the IBS group.

#### Criterion-related validity

We compared the scores of the 26-item and 18-item versions of the IBS-BRQ-J between the IBS patient, non-patient IBS, and control groups after adjusting for age to confirm the criterion-related validity (Table 2). The scores of both versions of the IBS-BRQ-J showed significant group differences, and the multiple comparison test showed that the IBS patient and non-patient IBS groups had higher scores than the control group and that the IBS patient group had a higher score than the non-patient IBS group.

#### Reliability

We calculated Cronbach’s α to estimate the reliability of the IBS-BRQ-J. The internal consistencies of the 26-item version and the 18-item version in all participants, the IBS group, and the control group were high (26-item version: all participants: α = 0.93, IBS group: α = 0.92, control group: α = 0.92; 18-item version: all participants: α = 0.92, IBS group: α = 0.91, control group: α = 0.91). Regarding the 18-item version, the internal consistencies of factor 1 (all participants: α = 0.92, IBS group: α = 0.91, control group: α = 0.93) and factor 2 (all participants: α = 0.83, IBS group: α = 0.77, control group: α = 0.79) were sufficient.

The item-total correlation analysis for the 26-item and 18-item versions of the IBS-BRQ-J (Table 6) showed that the correlations between each item and the total score were significant (26-item version: *ps* < 0.001, *rs* = 0.453–0.752; 18-item version: *ps* < 0.001, *rs* = 0.487–0.759).

**Table 6:** Correlations between the IBS-BRQ-J (26-item and 18-item version) and other variables

26 items		IBS-SI	Disruption to daily life (IBS-SI)	VSI	HADS (Anxiety)	HADS (Depression)	HADS (Total)	SASS
Total	*r*	0.556	0.538	0.598	0.316	0.233	0.302	-0.269
	*p*	<0.001	<0.001	<0.001	<0.001	<0.001	<0.001	<0.001
IBS group	*r*	0.488	0.464	0.518	0.449	0.386	0.454	-0.290
	*p*	<0.001	<0.001	<0.001	<0.001	<0.001	<0.001	0.004
Control group	*r*	0.452	0.461	0.518	0.113	0.127	0.133	-0.082
	*p*	<0.001	<0.001	<0.001	0.229	0.177	0.159	0.396
18 items		IBS-SI	Disruption to daily life (IBS-SI)	VSI	HADS (Anxiety)	HADS (Depression)	HADS (Total)	SASS
Total	*r*	0.560	0.533	0.610	0.305	0.240	0.300	-0.262
	*p*	<0.001	<0.001	<0.001	<0.001	<0.001	<0.001	<0.001
IBS group	*r*	0.491	0.464	0.533	0.434	0.399	0.452	-0.282
	*p*	<0.001	<0.001	<0.001	<0.001	<0.001	<0.001	0.005
Control group	*r*	0.450	0.442	0.521	0.098	0.131	0.126	-0.061
	*p*	<0.001	<0.001	<0.001	0.300	0.166	0.181	0.524

*IBS* irritable bowel syndrome

*IBS-BRQ-J* Japanese version of Irritable Bowel Syndrome-Behavioral Responses Questionnaire

*IBS-SI* IBS Severity Index

*VSI* Visceral Sensitivity Index

*HADS* Hospital Anxiety and Depression Scale

*SASS* Social Adaptation Self-evaluation Scale

## Discussion

### Study 1 (validity and reliability of the CS-FBD-J)

While the CS-FBD-J had a one-factor structure in common with the original version, items 6 and 18 of the Japanese version had low factor loadings and were deleted from this version. For the original version of the CS-FBD, although factor loadings for these two items were below 0.4, the authors determined to retain them based on their relevance to a priori themes (Toner et al., 1998). However, in the present study, we could find no reason to retain these items. Because the automatic thoughts referenced in these two items could not be interpreted by some people as being related to abdominal symptoms, the factor loadings of the two items were lower in this study (Table 3).

We confirm that the construct validity of the CS-FBD-J is sufficient. Regardless of the presence or absence of IBS in the participants, the CS-FBD-J and IBS symptom-related scales (IBS-SI and VSI) were positively correlated. Thus, the CS-FBD-J was shown to reflect gastrointestinal symptom-specific cognition. In contrast, general dysfunctional cognition not specific to gastrointestinal symptoms (DAS-24) was not prominently correlated with CS-FBD-J. Although this result differs from the original version, it may indicate a difference between general dysfunctional cognition and IBS-specific cognition. The difference in correlation coefficients between the CS-FBD-J and DAS-24 in the IBS group was slight in the 25-item and 23-item versions. This difference may relate to the finding that the DAS-24 scores were not significantly different in the presence of IBS. In addition, anxiety and depression (HADS) were correlated with CS-FBD-J only in the IBS group. Cognition of abdominal symptoms has been reported to be associated with anxiety and depression in IBS patients [3, 12], and the CS-FBD-J may reflect cognition specific to gastrointestinal symptoms that affect the emotions of individuals with IBS. Thus, the CS-FBD-J could be beneficial exclusively for individuals experiencing abdominal symptoms. In both the previous study [12] and the present data, the VSI was correlated with anxiety but not with depression, whereas the CS-FBD-J was correlated not only with anxiety but also with depression. Therefore, the CS-FBD can be distinguished from the concept measured by the VSI in that it reflects cognitions that affect both anxiety and depression. The CS-FBD and the VSI can be used for different purposes or can be combined to elaborate on cognitions related to gastrointestinal symptoms.

Sufficient criterion-related validity of the CS-FBD-J was also confirmed. The CS-FBD-J scores were higher in IBS patients, non-IBS patients, and the control group, in that order. In this study, the intensity of IBS symptoms (IBS-SI) by group was also in this order. This suggests that there is a large difference in CS-FBD-J scores, not only in the presence or absence of IBS but also in the high- and low-severity groups.

The high reliability of the CS-FBD-J was confirmed. The alpha coefficient was greater than 0.9, indicating a high internal consistency. The item-total correlation was significant for all items and exceeded 0.4 for the 23-item version.

### Study 2 (validity and reliability of the IBS-BRQ-J)

As in the original version, two factors were extracted: factor 1 consisted mainly of items related to avoidance of abdominal symptoms and associated difficulties, and factor 2 consisted mainly of items related to behavior obsessed with abdominal symptoms. The items comprising each factor were similar to those in the original version. However, the names of these factors in the original version of the IBS-BRQ were “avoidance behavior” and “control behavior,” but because items related to eating behavior for the purpose of controlling defecation were deleted, the content was closer to “obsession” than “control.” In the Japanese version, eight items with low factor loadings were deleted from the 26 items of the original version of the IBS-BRQ. This suggests that there may be cultural differences in the behavioral responses associated with abdominal symptoms. However, confirmatory factor analysis of the IBS-BRQ-J, which consists of 18 items and two factors, showed that the model fit was not satisfactory. Therefore, the factorial validity of the IBS-BRQ-J is unstable and needs to be further investigated.

In contrast, the construct validity and criterion-related validity of the IBS-BRQ-J were adequate, and correlations between the IBS-BRQ-J and general measures not related to IBS were significant only in the IBS group. The correlations between the IBS-BRQ-J and general scales not related to IBS were significant only in the IBS group, suggesting that negative emotions and maladjustment in people with IBS are likely to be symptom-driven, which explains the difference between the correlations in the IBS and control groups. Therefore, the results suggest that the IBS-BRQ-J may be an appropriate tool for assessing behavioral responses specific to IBS. Additionally, the IBS-BRQ-J would benefit only those suffering from abdominal symptoms, not healthy individuals, as the CS-FBD-J is.

The reliability of the IBS-BRQ-J was shown to be sufficiently high by the alpha coefficient and the item-total test; the alpha coefficient of factor 2 (behavior obsessed with abdominal symptoms) was lower because of a smaller number of items (seven items), but the reliability of both the IBS and control groups exceeded 0.7, and thus the reliability was considered to be sufficient.

Although the IBS-BRQ-J needs further study for the model fit of the two-factor structure, the results of other reliability and validity indices were sufficient. Currently, there is no other tool for assessing behavioral responses related to IBS other than the IBS-BRQ. The IBS-BRQ-J is expected to be an extremely useful and indispensable tool for research examining the psychological aspects of IBS and for assessment in the clinical practice of IBS using cognitive-behavioral therapy.

### The study limitations

The present study has several limitations. First, there were few patients with IBS among the participants in this study. In the previous studies of the original versions, all participants were patients with IBS (For the CS-FBD, 3 of 75 participants had other functional bowel disorders). As shown by the data of this study, the severity of IBS and non-IBS patients may differ. If we collect data from a sufficient number of patients with IBS or FBD and conduct factor analysis, the results may be different from those of this study. Second, in the assessment of the presence of IBS, the diagnostic criteria for IBS used in this study were not the latest Rome-IV criteria, which had not been published at the time the study started. In the latest diagnostic criteria, the presence of abdominal pain is essential for the diagnosis of IBS. Based on the above issues, further validation of CS-FBD-J and IBS-BRQ is required by extracting individuals with IBS using the latest diagnostic criteria and collecting sufficient data from IBS patients. Third, for the CS-FBD-J and IBS-BRQ-J, the number of items in the original and Japanese versions do not match, making a strict comparison with previous studies difficult. Fourth, the VSI used for construct validity is not a cognition-specific scale. Because we were unable to verify the significant correlation between the CS-FBD-J and the DAS-24 in the present study, we could not rigorously confirm whether the CS-FBD-J adequately measures the concept of cognition. Fifth, we did not determine the coexistence of organic gastrointestinal diseases among the study participants.

## Conclusion

The CS-FBD-J and IBS-BRQ-J are useful scales for assessing non-functional cognition and behavioral problems specific to gastrointestinal symptoms. The present study confirmed the reliability and validity of the 23-item version of the CS-FBS after the deletion of two items with low factor loadings. Regarding the IBS-BRQ-J, eight items with low factor loadings were deleted from the 26 items of the original version, and two factor structures were confirmed: Factor 1 consisted mainly of items related to behavior obsessed with abdominal symptoms, and factor 2 consisted mainly of items related to avoidance of abdominal symptoms and associated difficulties. Although the IBS-BRQ-J needs further study for the model fit of the two-factor structure, the results of other reliability and validity indices were sufficient. These scales are expected to contribute to a more precise assessment of patients in research on and clinical practice of functional gastrointestinal disorders.

## Supplementary Information

**Additional file 1:** **Appendix 1.** Japanese version of the CS-FBD.

**Additional file 2:** **Appendix 2.** Japanese version of the IBS-BRQ.

## Data Availability

We are not able to share our data because sharing data is not approved by our ethics committees.

## Abbreviations

IBS: Irritable bowel syndrome
CS-FBD-J: Japanese version of Cognitive Scale for Functional Bowel Disorders
IBS-BRQ-J: Japanese version of Irritable Bowel Syndrome-Behavioral Responses Questionnaire
IBS-SI: IBS Severity Index
VSI: Visceral Sensitivity Index
DAS-24: 24-item Dysfunctional Attitudes Scale
HADS: Hospital Anxiety and Depression Scale
SASS: Social Adaptation Self-evaluation Scale
