# Supplementary material for: Validity and reliability of the Japanese versions of cognitive and behavioral scales for irritable bowel syndrome
Source: Biopsychosoc Med. 2022 Jul 23;16:15. doi: 10.1186/s13030-022-00244-3 (PMC9308329; doi:10.1186/s13030-022-00244-3)
Supplement: Supplementary file 1 — Additional file 1: Appendix 1. Japanese version of the CS-FBD. [file 13030_2022_244_MOESM1_ESM.docx]

**Appendix 1** Japanese version of the CS-FBD

Underlined items: Items 6 and 18 were excluded from the Japanese version of the CS-FBD because of low factor loadings (< 0.40).
