# Supplementary material for: Validity and reliability of the Japanese versions of cognitive and behavioral scales for irritable bowel syndrome
Source: Biopsychosoc Med. 2022 Jul 23;16:15. doi: 10.1186/s13030-022-00244-3 (PMC9308329; doi:10.1186/s13030-022-00244-3)
Supplement: Supplementary file 2 — Additional file 2: Appendix 2. Japanese version of the IBS-BRQ. [file 13030_2022_244_MOESM2_ESM.docx]

Appendix 2 Japanese version of the IBS-BRQ

Underlined items: Items 1, 2, 8, 9, 10, 11, 23, and 24 were excluded from the Japanese version of the IBS-BRQ because of low factor loadings (< 0.40).
